# From theoretical energy to practical utilization: interfacial stability, transport kinetics, and cell-level design in high-energy lithium-metal batteries

**DOI:** 10.3389/fchem.2026.1870974

**Published:** 2026-06-16

**Authors:** Xianzheng Liu, Bangsheng Yin, Guangyuan Wang, Haotian Wu, Xiangjun Ren

**Affiliations:** 1 College of Mechanical Engineering, Shandong Huayu University of Technology, Dezhou, Shandong, China; 2 Department of Mechanical and Manufacturing Engineering, Faculty of Engineering and Built Environment, Universiti Kebangsaan Malaysia, Bangi, Selangor, Malaysia

**Keywords:** cathode interface, high-loading electrodes, lithium-metal batteries, pouch cells, practical energy density, transport kinetics

## Abstract

Lithium-metal batteries are widely regarded as one of the most promising routes toward cell-level energy densities beyond the limit of conventional graphite-based lithium-ion batteries. However, the practical energy delivered by a lithium-metal cell is not determined only by the theoretical capacity of the cathode or the low potential of lithium metal. In many cases, the accessible energy is gradually consumed by voltage decay, interfacial impedance growth, parasitic reactions, inactive component weight and incomplete utilization of thick electrodes. This review therefore focuses on the conversion of theoretical energy into practically deliverable full-cell energy rather than providing a broad survey of all high-energy lithium-metal battery chemistries. High-voltage layered oxides are discussed as representative systems for voltage preservation and cathode interfacial stabilization, while selected conversion cathodes are used to illustrate how sluggish reaction kinetics, mass transport limitations, and lean-electrolyte constraints reduce the utilization of high theoretical capacity. Lithium-metal anodes and pouch-cell design are further analyzed from the viewpoint of lithium inventory management and full-cell mass efficiency. By linking interfacial stability, transport kinetics, lithium reversibility, and cell-level design, this review emphasizes that practical energy density should be evaluated by retained capacity, average discharge voltage, electrolyte-to-capacity ratio, negative-to-positive electrode capacity ratio, areal capacity, and full-cell specific energy under realistic testing conditions.

## 1 Introduction

The continuous development of electric vehicles, portable electronics, unmanned aerial systems, and aerospace devices has created a strong demand for rechargeable batteries with higher specific energy, longer cycle life, and improved safety. Conventional lithium-ion batteries based on graphite anodes have achieved great commercial success because of their balanced electrochemical performance, mature manufacturing process, and acceptable safety. However, their practical energy density is gradually approaching the upper limit imposed by graphite anodes and conventional intercalation-type cathodes. Therefore, lithium-metal batteries have attracted renewed attention as promising candidates for next-generation high-energy battery systems. Recent studies have emphasized that the practical feasibility of lithium-metal batteries should be evaluated under realistic full-cell conditions rather than by material-level capacity alone. Key parameters such as cathode loading, areal capacity, electrolyte amount, lithium thickness, N/P ratio, E/C ratio, inactive component mass, and pouch-cell specific energy strongly affect whether high theoretical energy can be converted into practically deliverable energy (Chen et al., 2019; Liu et al., 2019; Xu et al., 2025).

Lithium metal is attractive because it has an ultrahigh theoretical specific capacity and the lowest electrochemical potential among common anode materials. Compared with graphite anodes, replacing graphite with lithium metal can substantially reduce the negative-electrode mass and, when properly paired with a suitable cathode and electrolyte, may slightly increase the thermodynamic cell voltage because of its lower anode potential. However, the practically delivered average voltage is ultimately governed by cathode voltage stability, interfacial polarization, lithium reversibility, and full-cell operating conditions. Therefore, the major practical advantage of lithium metal should be understood as reducing anode mass and enabling higher cell-level specific energy, rather than simply increasing the full-cell voltage (Qian et al., 2018; Ren et al., 2019). However, the use of lithium metal alone does not guarantee a practical high-energy battery. The total energy output of a lithium-metal cell is determined not only by the theoretical capacity of the active materials, but also by the stability of the cathode, reversibility of the lithium-metal anode, electrolyte compatibility, electrode loading, inactive component mass and cell configuration (Wang et al., 2020; Zhao et al., 2021; Zhao et al., 2024). In many laboratory studies, impressive capacities are obtained under conditions that are not compatible with practical cells, such as low cathode loading, excess electrolyte, thick lithium foil and small-area coin-cell geometry. These conditions may hide degradation mechanisms that become severe in pouch cells.

For this reason, it is necessary to distinguish between theoretical energy density and practical usable energy. Theoretical energy density is usually calculated from the specific capacity and operating voltage of electrode materials. Practical usable energy, in contrast, refers to the energy that a full cell can repeatedly deliver under realistic conditions, including high areal loading, lean electrolyte, limited lithium excess and controlled inactive mass. This difference is especially important for lithium-metal batteries. A cathode with high capacity may still fail to provide high usable energy if it suffers rapid voltage decay, oxygen release, particle cracking, sluggish transport or severe interfacial side reactions. Similarly, a lithium-metal anode may support long cycling in the presence of excessive lithium, but such an anode configuration reduces the practical energy density of the entire cell.

Therefore, the central question in high-energy lithium-metal batteries is not simply how to obtain higher theoretical capacity, but how to retain electrochemical energy as practically deliverable full-cell output. In this review, practical deliverable energy is understood as the energy that can be repeatedly extracted from a full cell after considering capacity utilization, average discharge voltage, interfacial impedance, lithium inventory, electrolyte amount, electrode loading, and inactive component mass. To keep the discussion focused, this mini-review does not aim to comprehensively summarize all lithium-metal battery chemistries. Instead, representative cathode systems are selected according to the dominant energy-loss mechanisms they illustrate.

High-voltage layered oxides, including LiCoO_2_, Ni-rich oxides, and Li-rich oxides, are discussed mainly to show how voltage decay, oxygen release, surface reconstruction, and interfacial impedance growth reduce usable energy. Conversion-type cathodes, represented by sulfur and oxygen cathodes, are discussed not as independent broad topics, but as examples of systems in which high theoretical capacity is difficult to translate into high areal energy under lean-electrolyte and high-loading conditions. The lithium-metal anode is analyzed in terms of lithium inventory loss, dead lithium formation, and the mass penalty associated with excessive lithium. Finally, pouch-cell design is considered as the cell-level step that determines whether retained electrochemical energy can be converted into practical gravimetric and volumetric energy density. Through this organization, the review uses different material systems to support one central theme: theoretical energy can become practical energy only when voltage stability, interfacial compatibility, transport kinetics, lithium reversibility, and inactive-mass control are optimized together.

**FIGURE 1:**
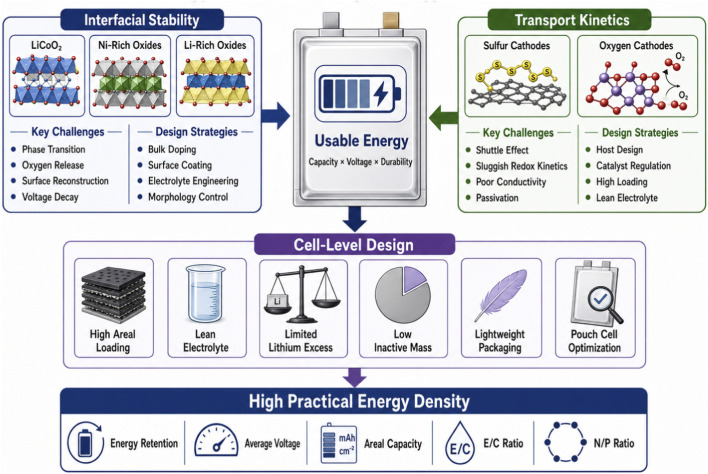
Schematic illustration of practical energy utilization in high-energy lithium-metal batteries.

As summarized in Figure 1, practical energy utilization in lithium-metal batteries depends on the coordinated optimization of interfacial stability, transport kinetics and cell-level design. These factors jointly determine energy retention, average voltage, areal capacity, E/C ratio and N/P ratio, which are more relevant to practical high-energy cells than material-level capacity alone.

To avoid ambiguity, the key practical metrics used in this review are defined here. Areal capacity refers to the discharge capacity normalized to electrode area, commonly expressed as mAh cm^-2^, and reflects whether a cathode can provide sufficient energy per unit cell footprint. Average discharge voltage is the capacity-weighted mean voltage during discharge and directly determines the actual energy output together with capacity. Energy retention is the retained discharge energy after cycling, usually expressed as a percentage of the initial discharge energy; it is more informative than capacity retention alone because it includes both capacity fading and voltage decay. The electrolyte-to-capacity ratio, abbreviated as E/C ratio, describes the amount of electrolyte normalized to cell capacity and is commonly expressed as g Ah^-1^ or μL mAh^-1^. A low E/C ratio is essential for high practical energy density because electrolyte is an inactive mass component, although excessively lean electrolyte may cause poor wetting, severe polarization, and accelerated interfacial degradation. The negative-to-positive electrode capacity ratio, abbreviated as N/P ratio, represents the ratio between the available negative-electrode capacity and positive-electrode capacity. For lithium-metal cells, this value is mainly determined by lithium thickness and cathode areal capacity. A lower N/P ratio reduces excess lithium mass and improves gravimetric energy density, but it also makes the cell more sensitive to irreversible lithium loss and unstable solid-electrolyte interphase formation.

Beyond these definitions, the key metrics are linked through a continuous energy-loss chain. Cathode interfacial instability at high voltage promotes electrolyte oxidation, transition-metal dissolution, gas generation, and cathode-electrolyte interphase growth. These processes consume electrolyte and active lithium, increase interfacial resistance, and reduce the average discharge voltage. The resulting impedance growth further intensifies transport polarization in high-loading electrodes, leading to incomplete active-material utilization and lower areal energy output. On the anode side, electrolyte-derived species and cathode crossover products can disturb SEI chemistry, accelerate dead lithium formation, and deplete the lithium inventory. At the pouch-cell level, these losses often require compensation by excess electrolyte, thicker lithium foil, or additional protective components, which increases inactive mass and decreases practical specific energy. Therefore, interfacial stability, transport kinetics, lithium inventory, and cell-level design should be regarded as a coupled energy-retention chain rather than independent optimization targets.

## 2 High-voltage layered cathodes: preserving voltage as usable energy

Layered oxide cathodes remain among the most realistic choices for high-energy lithium-metal batteries because they combine high operating voltage, relatively mature synthesis, high tap density and good compatibility with existing electrode-manufacturing technologies. Compared with conversion-type cathodes, layered oxides generally show higher average discharge voltage and better volumetric energy density. However, their energy advantage can be preserved only when structural stability and interfacial compatibility are maintained during repeated cycling. Once the cathode loses its high-voltage plateau or experiences rapid impedance growth, the actual energy output of the full cell decreases even if the measured capacity remains relatively high.

LiCoO_2_ is a representative high-voltage layered oxide cathode. It has been widely used in commercial lithium-ion batteries because of its high density, stable layered structure and reliable processing behavior. Increasing the upper cut-off voltage of LiCoO_2_ can extract more lithium and increase the discharge capacity, making it attractive for high-energy applications (Sun et al., 2023; Zhuang et al., 2023). However, deep delithiation at high voltage induces several coupled degradation processes. First, the layered structure undergoes irreversible phase transitions, accompanied by lattice contraction and internal stress accumulation. Second, lattice oxygen becomes more active, resulting in oxygen release and surface reconstruction. Third, the highly oxidized cathode surface accelerates electrolyte decomposition, leading to cathode-electrolyte interphase growth and impedance increase. These processes reduce both capacity and average discharge voltage, which directly weakens the usable energy of the cell.

Recent studies suggest that the stability of high-voltage LiCoO_2_ depends strongly on both bulk structure and particle morphology. If lithium extraction occurs unevenly inside the particle, local stress develops and promotes microcrack formation. These cracks expose fresh surfaces to the electrolyte and provide new reaction sites, thereby accelerating surface degradation. Morphology-controlled LiCoO_2_ particles with more uniform lithium distribution can suppress stress concentration and delay structural failure (Zhang et al., 2025). This indicates that particle engineering should not be treated as a simple morphological optimization. Instead, it should be viewed as a strategy to regulate electrochemical reaction uniformity and preserve usable energy.

Mechanistically, morphology optimization contributes to voltage stability by reducing spatially uneven delithiation and suppressing local stress concentration. In high-voltage LiCoO_2_, nonuniform lithium extraction can generate heterogeneous states of charge within secondary particles, leading to anisotropic lattice contraction and intergranular cracking. Once cracks form, fresh internal surfaces are exposed to the electrolyte, which accelerates oxygen loss, electrolyte oxidation, CEI thickening, and impedance growth. These processes increase polarization and lower the average discharge voltage. Therefore, morphology-controlled particles with more homogeneous lithium transport and reduced internal strain help maintain the high-voltage plateau by delaying crack-induced surface degradation and preserving interfacial continuity.

Surface chemistry is equally important for high-voltage LiCoO_2_. Surface coatings, doping layers, artificial rock-salt or spinel phases and lattice-coherent interfacial layers have been developed to reduce oxygen loss and electrolyte attack (Cai et al., 2023; Qin et al., 2022; Zhang et al., 2023; Zhang A. et al., 2024). However, the protective layer must be carefully designed. A thick or poorly conductive coating may suppress side reactions but block lithium-ion transport, reducing rate performance and active-material utilization. An ideal cathode surface layer should provide four functions at the same time: suppress oxygen release, reduce electrolyte oxidation, maintain lithium-ion transport and accommodate interfacial strain. Therefore, the optimization target should not be simply “more stable surface”, but “transport-preserving interfacial stability”.

Surface coatings and doping layers stabilize voltage through different but complementary mechanisms. A surface coating can act as a chemical and physical barrier between the highly oxidized LiCoO_2_ surface and the electrolyte, thereby suppressing solvent oxidation, transition-metal dissolution, oxygen release, and continuous CEI growth. If the coating is ionically conductive and sufficiently thin, it can reduce parasitic reactions without severely blocking Li^+^ transport, thereby lowering interfacial polarization and helping retain the average discharge voltage. In contrast, doping or lattice-coherent interfacial layers mainly regulate the near-surface crystal structure and electronic environment. Properly selected dopants can strengthen transition-metal–oxygen bonding, reduce surface oxygen activity, suppress irreversible layered-to-rock-salt or spinel-like reconstruction, and mitigate lattice strain during deep delithiation. These effects help preserve the high-voltage reaction pathway and reduce voltage decay. Therefore, the practical role of coating and doping should be understood not only as improving capacity retention, but also as maintaining voltage output by slowing oxygen-related degradation, electrolyte decomposition, and impedance accumulation.

Recent literature further confirms that high-voltage LiCoO_2_ degradation should be understood as a coupled process involving surface oxygen instability, bulk phase transition, particle-level stress accumulation, electrolyte oxidation, and interfacial impedance growth (Lin et al., 2026). Therefore, morphology regulation, surface reconstruction control, coating/doping strategies, and electrolyte-compatible interphase design should be evaluated not only by capacity retention but also by average-voltage retention and full-cell energy retention.

Ni-rich layered oxide cathodes offer higher specific capacity than conventional layered oxides because the Ni redox couple contributes large reversible capacity. Increasing the Ni content can improve the energy density of the cathode, but it also intensifies intrinsic structural and interfacial problems (Wang et al., 2004; Yang et al., 2024). One major issue is Li/Ni cation mixing. Because Ni^2+^ has an ionic radius close to that of Li^+^, Ni ions can occupy lithium layers and hinder lithium-ion diffusion. This cation disorder reduces accessible capacity and increases polarization. The degree of cation mixing is strongly affected by synthesis conditions, including lithium stoichiometry, calcination temperature and oxygen atmosphere (Chen et al., 2024; Tayal et al., 2024). Therefore, synthesis control is directly linked to practical energy density.

Surface degradation in Ni-rich cathodes is another severe challenge. During charging, highly oxidized Ni-rich surfaces are chemically reactive and prone to oxygen evolution, electrolyte decomposition and surface reconstruction. The reconstructed surface layer often becomes electrochemically sluggish, which limits lithium transport and increases interfacial resistance. In practical pouch cells, this problem is amplified by high areal loading and lean electrolyte because transport limitations become more pronounced. Gradient doping, concentration-gradient particles, surface reconstruction and electrolyte additives have been used to stabilize Ni-rich cathodes (Cui et al., 2024; Luo et al., 2022; Zhu et al., 2023). Nevertheless, these strategies should be evaluated not only by half-cell capacity retention, but also by full-cell energy retention, average voltage and impedance growth under realistic conditions.

Li-rich layered oxides represent another promising family of high-capacity cathodes. Their high capacity originates from both transition-metal redox and oxygen redox reactions (Cui et al., 2025; Li et al., 2018). However, their practical use is limited by voltage hysteresis, voltage decay and irreversible oxygen-related structural evolution. These problems are particularly damaging to practical energy density because energy is determined by both capacity and voltage. A Li-rich cathode may retain a large fraction of its capacity but still suffer from significant energy loss if the average discharge voltage continuously decreases.

The key issue in Li-rich oxides is how to use oxygen redox reversibly without triggering long-term structural instability. Irreversible oxygen redox can promote transition-metal migration and layered-to-spinel or rock-salt transformation, leading to voltage decay (Myeong et al., 2018; Radin et al., 2019; Zhang M. et al., 2024). Therefore, Li-rich cathodes should be evaluated by energy retention rather than capacity retention alone. Strategies such as local structure regulation, surface protection, defect engineering and cation doping should be designed to stabilize oxygen participation while preventing structural collapse. For lithium-metal full cells, this challenge is even more important because voltage decay on the cathode side combines with lithium loss on the anode side, causing accelerated full-cell performance degradation.

These examples show that voltage preservation is controlled by both structural and interfacial mechanisms. For LiCoO_2_, morphology regulation, surface coatings, and doping layers mainly aim to suppress crack formation, oxygen release, electrolyte oxidation, and CEI-induced polarization. For Ni-rich cathodes, reducing cation mixing, surface reconstruction, and interfacial impedance is essential for maintaining Li^+^ transport and average voltage. For Li-rich cathodes, stabilizing oxygen redox and preventing irreversible transition-metal migration are necessary to reduce voltage hysteresis and long-term voltage decay. Therefore, the section theme of “preserving voltage as usable energy” is not limited to maintaining the nominal operating voltage; rather, it refers to retaining the discharge-energy output by simultaneously controlling bulk structural evolution, surface chemistry, and interfacial transport resistance.

Overall, high-voltage layered cathodes demonstrate that preserving voltage is as important as preserving capacity. For lithium-metal batteries targeting high practical energy density, cathode design should focus on stable high-voltage operation, controlled surface chemistry and suppressed impedance growth. The usable energy of these systems depends on whether the cathode can maintain high average voltage and sufficient capacity under realistic full-cell constraints.

## 3 Interfacial chemistry between cathodes and electrolytes

The cathode-electrolyte interface is a critical region where material-level instability is converted into cell-level energy loss. At high voltage, electrolyte oxidation, gas generation, transition-metal dissolution and cathode surface reconstruction occur simultaneously. These reactions consume active lithium and electrolyte, increase cell impedance and reduce the effective operating voltage. For lithium-metal batteries, interfacial side reactions are more serious because the lithium-metal anode is highly sensitive to electrolyte composition and lithium inventory loss. Decomposition products generated at the cathode can migrate through the electrolyte and affect the lithium-metal surface, forming a coupled cathode-anode degradation pathway.

A stable cathode-electrolyte interphase is therefore necessary for high-energy lithium-metal batteries. However, a useful interphase should not simply be chemically inert. It must allow fast lithium-ion transport and remain mechanically stable during volume changes or surface reconstruction. Electrolyte additives can promote the formation of inorganic-rich interphases, while highly concentrated or localized high-concentration electrolytes can change solvation structures and suppress solvent oxidation. Fluorinated solvents and lithium salts have also been widely explored to improve oxidative stability and promote LiF-containing interphase chemistry. When properly designed, these electrolyte formulations may contribute to stabilizing both high-voltage cathode surfaces and lithium-metal anodes by reducing solvent activity, modifying decomposition pathways, and forming more inorganic-rich CEI/SEI layers. However, their effectiveness is strongly dependent on salt concentration, fluorinated-solvent structure, cathode operating voltage, lithium-metal reversibility, temperature, and lean-electrolyte conditions. Therefore, fluorinated electrolyte design should be evaluated under practical full-cell conditions rather than assumed to universally stabilize both interfaces (Zhang A. et al., 2024).

Nevertheless, electrolyte engineering involves trade-offs. A highly stable electrolyte may have high viscosity, poor wettability or limited low-temperature performance. A fluorinated electrolyte may improve interfacial stability but increase cost and reduce environmental compatibility. A thick cathode-electrolyte interphase may suppress parasitic reactions but increase charge-transfer resistance. Therefore, electrolyte design should be evaluated under practical cell conditions rather than only by linear sweep voltammetry or low-loading half-cell cycling. Important parameters include electrolyte amount, electrode loading, cell format, current density and long-term impedance evolution.

For high-voltage layered cathodes, the cathode-electrolyte interface should be designed together with cathode surface engineering. If the cathode surface coating is incompatible with the electrolyte-derived interphase, cracks or nonuniform interphase growth may occur. Conversely, a coherent surface layer that guides stable interphase formation can reduce oxygen release, transition-metal dissolution and electrolyte oxidation. This combined design is more promising than treating cathode modification and electrolyte formulation as separate topics.

The effect of cathode interfacial instability is not confined to the cathode side. Electrolyte oxidation at highly charged cathode surfaces continuously consumes solvent and salt, while transition-metal ions and soluble oxidation products may migrate toward the lithium-metal anode. These crossover species can disturb SEI formation, promote parasitic lithium consumption, and accelerate dead lithium accumulation. As lithium inventory decreases, the full cell becomes more vulnerable to capacity loss, voltage polarization, and rapid energy decay, especially under low-N/P and lean-electrolyte conditions. Therefore, cathode interfacial stabilization should be considered not only as a cathode-protection strategy, but also as a cell-level approach to reducing electrolyte consumption, maintaining lithium inventory, suppressing impedance growth, and preserving pouch-cell energy density.

## 4 Conversion cathodes: high theoretical capacity under transport constraints

Conversion cathodes are included in this review not to provide a comprehensive survey of lithium-sulfur or lithium-oxygen batteries, but to highlight a critical gap between theoretical capacity and practical energy delivery. Unlike layered oxide cathodes, whose practical energy loss is often dominated by voltage decay and interfacial degradation, conversion cathodes usually suffer from incomplete reaction, sluggish redox kinetics, product passivation, poor mass transport, and strong dependence on electrolyte amount. These limitations become particularly severe under practical testing conditions, including high areal loading, lean electrolyte, limited lithium excess, and pouch-cell-level mass constraints. Therefore, sulfur and oxygen cathodes are discussed here as representative examples showing that high theoretical capacity can contribute to practical energy density only when reaction kinetics, transport pathways, and inactive mass are simultaneously controlled.

Sulfur cathodes are attractive for lithium-metal batteries because sulfur has high theoretical capacity, low cost and natural abundance. Lithium-sulfur batteries have the potential to achieve high gravimetric energy density, especially when paired with lithium metal. However, the conversion reaction of sulfur is far more complex than the intercalation reaction of layered oxides. During discharge, sulfur is converted through a series of soluble polysulfide intermediates and finally forms insoluble Li_2_S. These intermediates can migrate between electrodes, causing the well-known shuttle effect. In addition, both sulfur and Li_2_S have poor electronic conductivity, which limits active-material utilization (Pang et al., 2018; Xue et al., 2019).

From the perspective of practical energy utilization, the main challenge of sulfur cathodes is not merely achieving high capacity in a small coin cell. The real challenge is to maintain high areal capacity under high sulfur loading, lean electrolyte, high sulfur fraction, and limited lithium excess. Recent practical and benchmarking studies indicate that Li-S cells should be evaluated by coupled parameters such as sulfur loading, areal capacity, electrolyte-to-sulfur ratio, electrolyte-to-capacity ratio, lithium excess, active-material fraction, cycle life, and pouch-cell specific energy (Nandagiri et al., 2026; Shakil et al., 2026; Yari et al., 2025). Porous carbon, polar compounds, metal compounds, catalytic hosts, and framework structures can confine polysulfides and accelerate redox conversion, but these components also introduce inactive mass. Therefore, sulfur cathode design must balance polysulfide confinement, catalytic activity, electronic/ionic transport, electrolyte accessibility, and inactive-mass control.

Lean electrolyte is another crucial factor for practical lithium-sulfur batteries. A large amount of electrolyte can improve sulfur utilization and reduce polarization, but it substantially reduces cell-level energy density. Under lean-electrolyte conditions, ion transport becomes difficult, polysulfide conversion becomes incomplete and electrode passivation becomes more severe. Therefore, sulfur cathodes need architectures that can maintain electrolyte accessibility and reaction uniformity without excessive porosity. High sulfur loading and high sulfur fraction should be developed together with catalytic host design and electrolyte optimization.

Lithium-oxygen batteries have an even higher theoretical energy density because oxygen can be supplied from outside the cell or stored in a porous cathode. However, their practical realization is limited by oxygen reduction/evolution kinetics, discharge-product accumulation, electrolyte instability and cathode passivation (Zor et al., 2024). During discharge, insoluble products such as Li_2_O_2_ form in the cathode pores and block transport pathways. During charge, decomposition of these products requires high overpotential, which accelerates electrolyte degradation and reduces round-trip efficiency. Therefore, the oxygen cathode must provide gas diffusion, electron transport, ion transport and catalytic activity at the same time.

The practical energy density of lithium-oxygen batteries is therefore governed by architecture and reversibility rather than theoretical oxygen capacity alone. A highly porous cathode may store more discharge products, but excessive porosity reduces volumetric energy density and mechanical robustness. A highly active catalyst may reduce overpotential, but it may also promote electrolyte decomposition. Therefore, oxygen cathode design should focus on reversible product formation and decomposition, stable catalyst-electrolyte interfaces and controlled pore structures. In addition, realistic evaluation should consider oxygen management, moisture and carbon dioxide sensitivity, cell sealing and safety.

Recent Li-O_2_ studies further indicate that practical evaluation should include not only theoretical oxygen-related capacity but also areal capacity, electrolyte amount, round-trip efficiency, discharge-product reversibility, oxygen transport, lithium-metal corrosion, and stacked-cell or pouch-cell-level energy-density assessment (Matsuda et al., 2024; Sandhiya and Elumalai, 2026). These references support the view that the practical translation of Li-O_2_ batteries is limited by the coupled instability of the lithium-metal anode, air cathode, electrolyte, and oxygen-related intermediates rather than by cathode capacity alone.

Therefore, the practical relevance of conversion cathodes should not be judged by theoretical capacity or low-loading coin-cell capacity alone. Instead, they should be evaluated by whether high areal capacity, high active-material fraction, low electrolyte-to-capacity ratio, stable average discharge voltage, and acceptable cycle life can be achieved simultaneously under realistic full-cell conditions. Without these coupled metrics, high material-level capacity cannot be reliably translated into practically deliverable energy.

## 5 Lithium-metal anode and lithium inventory management

The lithium-metal anode is the foundation of lithium-metal batteries, but it is also one of the main sources of practical energy loss. During repeated plating and stripping, lithium may form dendritic or porous deposits, leading to increased surface area and continuous electrolyte consumption. Part of the lithium becomes electrically isolated and forms dead lithium. Meanwhile, unstable solid-electrolyte interphase formation consumes both electrolyte and active lithium. These reactions reduce Coulombic efficiency and gradually deplete the lithium inventory (Qian et al., 2018).

In many laboratory studies, thick lithium foil is used to compensate for lithium loss and extend cycle life. However, thick lithium foil significantly increases cell mass and lowers practical energy density. Therefore, the N/P ratio and lithium thickness must be carefully controlled when evaluating high-energy lithium-metal batteries. A cell with excessive lithium may show stable cycling but fail to meet realistic energy-density targets. In contrast, a cell with limited lithium excess better reflects practical requirements but is more sensitive to parasitic reactions.

Stabilizing the lithium-metal interface requires electrolyte-derived SEI control, current-density regulation and uniform lithium-ion flux. Electrolyte formulation is particularly important because it determines the composition and mechanical properties of the SEI. Inorganic-rich SEI layers containing LiF or other stable components are often beneficial for suppressing continuous side reactions. Three-dimensional hosts and artificial interlayers can also improve lithium deposition uniformity. However, these strategies must be evaluated by their influence on total cell mass. A heavy host or thick interlayer may improve cycling but reduce energy density.

Lithium inventory management should therefore be integrated with cathode and pouch-cell design. High-loading cathodes require sufficient lithium supply, but excessive lithium reduces gravimetric energy density. Lean electrolyte reduces inactive mass, but it increases the demand for highly reversible lithium cycling. High-voltage cathodes may oxidize electrolyte and generate species that destabilize the lithium-metal anode. These couplings indicate that the lithium-metal anode cannot be optimized independently. Its practical performance should be evaluated in full cells with realistic cathode loading, limited electrolyte and controlled lithium excess.

## 6 Cell-level design and practical reporting standards

The transition from coin cells to pouch cells often exposes hidden energy-density losses. In small laboratory cells, excess electrolyte, thick lithium foil and oversized separators have limited influence on reported material capacity. In pouch cells, however, every inactive component contributes directly to total mass and volume. Current collectors, separators, tabs, packaging films and electrolyte may occupy a significant fraction of the cell weight. Therefore, a high-capacity cathode can fail to deliver high practical energy density if the cell requires too much electrolyte, lithium excess or structural support.

As summarized in Table 1, the practical energy utilization of lithium-metal batteries is governed by several coupled factors, including cathode stability, conversion-reaction kinetics, lithium-metal reversibility, electrolyte/interphase compatibility, and pouch-cell configuration. Therefore, practical high-energy lithium-metal batteries should be evaluated not only by material-level capacity but also by energy retention, average voltage, areal capacity, E/C ratio, N/P ratio, and full-cell specific energy.

**TABLE 1:** Key factors affecting practical energy utilization in high-energy lithium-metal batteries.

Key aspect	Main energy-loss pathway	Optimization focus	Practical evaluation metric
High-voltage layered cathodes	Phase transition, oxygen release, surface reconstruction, voltage decay	Bulk doping, surface coating, morphology control, cathode–electrolyte interphase regulation	Average voltage, energy retention, impedance growth
Conversion-type cathodes	Shuttle effect, sluggish redox kinetics, poor conductivity, cathode passivation	Host design, catalyst regulation, high loading, lean-electrolyte compatibility	Areal capacity, active-material fraction, E/C ratio
Lithium-metal anode	Dead lithium formation, dendrite growth, unstable SEI, irreversible lithium loss	Stable SEI formation, uniform Li plating/stripping, limited lithium excess	N/P ratio, Li thickness, coulombic efficiency
Electrolyte and interphase	Electrolyte oxidation, continuous side reactions, poor interfacial compatibility	High-voltage-stable electrolyte, additive design, interphase chemistry control	E/C ratio, ionic conductivity, interfacial resistance
Pouch-cell configuration	Excess inactive mass from separator, current collector, tabs, electrolyte, and packaging	Lightweight packaging, balanced electrode loading, full-cell mass optimization	Cell-level Wh kg^-1^, energy retention, inactive mass fraction

These metrics should be interpreted as coupled cell-level parameters rather than isolated descriptors. Areal capacity determines the amount of charge stored per unit electrode area, but high areal capacity can contribute to high practical energy only when the thick electrode remains sufficiently wetted and ionically accessible. Average discharge voltage converts capacity into energy; therefore, voltage decay in high-voltage or Li-rich cathodes can reduce practically deliverable energy even when the capacity retention appears acceptable. Energy retention should therefore be reported together with capacity retention, because it captures the combined effects of capacity loss, voltage decay, and impedance-induced polarization. The E/C ratio controls the mass penalty from electrolyte. Reducing the E/C ratio increases the fraction of active components in the cell, but it also increases the need for stable interfaces, continuous ion transport, and efficient electrolyte distribution. The N/P ratio controls the mass penalty and lithium-inventory buffer associated with the lithium-metal anode. Excessive lithium can mask interfacial instability and artificially prolong cycling, whereas limited lithium excess better reflects practical pouch-cell operation but requires higher Coulombic efficiency and more stable SEI chemistry. Therefore, practical lithium-metal batteries should be evaluated by the combined evolution of areal capacity, average voltage, energy retention, E/C ratio, N/P ratio, lithium thickness, and full-cell specific energy, as summarized in Table 2.

**TABLE 2:** Definitions and practical significance of key evaluation metrics for high-energy lithium-metal batteries.

Metric	Unit	Definition	Practical significance
Areal capacity	mAh cm^-2^	Discharge capacity normalized to electrode area	Determines whether the electrode can deliver sufficient energy per unit cell footprint; too low areal capacity may overestimate practical feasibility
Cathode loading	mg cm^-2^	Mass of cathode active material per electrode area	High loading helps reduce the relative contribution of inactive components, but may intensify ion-transport limitations and polarization
Average discharge voltage	V	Capacity-weighted mean discharge voltage	Directly converts capacity into energy; voltage decay reduces usable energy even if capacity retention remains high
Energy retention	%	Retained discharge energy after cycling relative to the initial discharge energy	Captures both capacity fading and voltage decay, making it more relevant than capacity retention alone for high-energy cells
E/C ratio	g Ah^-1^ or μL mAh^-1^	Electrolyte amount normalized to cell capacity	Lower E/C ratio reduces inactive electrolyte mass, but overly lean electrolyte can cause poor wetting, sluggish transport, and accelerated interfacial degradation
N/P ratio	Dimensionless	Ratio of negative-electrode capacity to positive-electrode capacity	Lower N/P ratio reduces excess lithium mass and improves practical energy density, but increases sensitivity to irreversible lithium loss
Lithium thickness	μm	Thickness of lithium-metal foil or lithium reservoir	Thick lithium can artificially improve cycling stability but lowers gravimetric energy density
Full-cell specific energy	Wh kg^-1^	Discharge energy normalized to total cell mass	Direct indicator of practical energy output at the cell level
Impedance evolution	Ω or % increase	Change in cell resistance during cycling	Impedance growth increases polarization, lowers average discharge voltage, and reduces energy efficiency

This cell-level energy loss can be understood as the accumulated consequence of coupled interfacial and transport degradation. Cathode degradation reduces average discharge voltage through oxygen loss, surface reconstruction, transition-metal migration, and interfacial impedance growth. Electrolyte consumption weakens ionic continuity and increases the practical demand for electrolyte reserve. Lithium inventory loss reduces reversible capacity and makes the cell more sensitive to local current-density fluctuation. Transport limitations in thick electrodes further reduce active-material utilization at practical current densities. When these losses are compensated by adding excess electrolyte, thick lithium, oversized separators, or heavy protective layers, the inactive mass fraction increases. As a result, pouch-cell specific energy decreases even if the material-level capacity remains high.

A practical pouch-cell design should begin with a target cell-level energy density and then work backward to determine the allowed mass of each component. For layered oxide cathodes, increasing areal loading and maintaining high average voltage are often more important than maximizing first-cycle capacity. For sulfur cathodes, reducing electrolyte amount and increasing sulfur loading are essential. For oxygen cathodes, stable air-electrode design and reversible discharge-product management are required. In all cases, the inactive mass fraction must be minimized without sacrificing safety or manufacturability.

Reporting standards need to be improved to make different studies comparable. In addition to specific capacity and cycle number, researchers should report cathode loading, areal capacity, electrode thickness, porosity, electrolyte amount, E/C ratio, lithium thickness, N/P ratio, separator type, current collector thickness, pouch-cell mass and testing pressure if applicable. Energy retention should be reported together with capacity retention because voltage decay can be hidden when only capacity is shown. Average discharge voltage and impedance evolution are especially important for Li-rich and high-voltage cathodes.

This reporting framework is consistent with recent analyses of practical lithium-metal cells and pouch-cell evaluation standards (Chen et al., 2019; Xu et al., 2025). These studies show that high capacity measured under excess-electrolyte, low-loading, or thick-lithium conditions may overestimate the practical feasibility of lithium-metal batteries. Therefore, cathode loading, areal capacity, electrolyte amount, E/C ratio, lithium thickness, N/P ratio, separator and current-collector mass, pouch-cell mass, average discharge voltage, and energy retention should be reported together to enable meaningful comparison among different studies.

Furthermore, realistic testing should include high areal capacity and lean-electrolyte conditions. Low-loading electrodes may show excellent rate capability because ion transport distances are short. However, thick electrodes experience stronger concentration gradients and more severe polarization. Similarly, abundant electrolyte can improve wetting and reduce transport resistance, but it is incompatible with high practical energy density. Therefore, practical evaluation must intentionally expose the limitations that appear in real pouch cells.

To provide a more explicit practical baseline, this review suggests that high-energy lithium-metal full cells should be evaluated, whenever possible, under cathode areal capacities of at least three mA h cm^-2^, lean electrolyte conditions with an E/C ratio not higher than 3 g Ah^-1^ or 3 μL mAh^-1^, and limited lithium excess with a lithium-metal thickness not higher than 50 μm. In addition, an N/P ratio not higher than two is suggested as a practical upper limit for lithium-metal full-cell evaluation, while lower N/P values should be pursued when stable lithium reversibility can be maintained. These values should not be interpreted as universal pass/fail criteria for all lithium-metal battery chemistries, because the optimal testing window depends on cathode type, electrolyte formulation, target energy density, safety margin, and cell format. Nevertheless, they provide a useful baseline for distinguishing practical full-cell evaluation from proof-of-concept testing based on low cathode loading, flooded electrolyte, or thick lithium foil. Therefore, areal capacity, E/C ratio, N/P ratio, lithium thickness, average discharge voltage, energy retention, impedance evolution, and full-cell specific energy should be reported together to determine whether theoretical energy is truly converted into practically deliverable energy.

## 7 Future perspectives for practical energy utilization

Future high-energy lithium-metal batteries should be designed around the concept of retained usable energy. First, cathode interfaces should be adaptive rather than simply passive. The ideal interface should suppress parasitic reactions while allowing fast lithium transport and tolerating repeated strain. For high-voltage layered oxides, this means combining bulk stabilization, surface reconstruction control and electrolyte compatibility. For Li-rich oxides, it means stabilizing oxygen redox while reducing voltage hysteresis and voltage decay.

Second, conversion cathodes should be developed under practical constraints from the beginning. Sulfur cathodes should be optimized at high sulfur loading, high sulfur fraction and lean electrolyte. Oxygen cathodes should be evaluated with realistic oxygen-management conditions and long-term reversibility. In both cases, high theoretical capacity should not be treated as sufficient evidence of practical potential. The key question is whether the system can sustain high areal energy output with limited inactive mass.

Third, lithium-metal anodes should be incorporated into the energy-density budget. Strategies that improve lithium reversibility are valuable only if they do not introduce excessive mass or volume. Thin lithium metal, stable SEI formation and electrolyte compatibility are necessary for practical pouch cells. The N/P ratio should be clearly reported, and cycling performance should be interpreted together with lithium excess. As a practical upper limit, N/P ≤ 2 is suggested for lithium-metal full-cell evaluation, while lower values should be pursued when stable lithium reversibility can be maintained. Similarly, cathode areal capacity, E/C ratio, lithium thickness, average discharge voltage, energy retention, and full-cell specific energy should be reported together to determine whether a given cell configuration is truly relevant to practical high-energy operation.

Finally, the field should move from single-component optimization toward integrated cell engineering. Cathode materials, electrolyte formulations, lithium-metal protection and pouch-cell configuration must be designed together. A modification that improves half-cell cycling may not be useful if it reduces tap density, increases inactive mass or requires impractical electrolyte amounts. Conversely, a moderate improvement in interfacial stability may be highly valuable if it enables higher loading, lower electrolyte content or thinner lithium foil. Therefore, the next breakthrough in lithium-metal batteries will likely come from integrated material-cell design rather than isolated material discovery.

## 8 Conclusion

Lithium-metal batteries provide an attractive route toward high-energy rechargeable batteries, but their practical performance cannot be judged solely by theoretical capacity or first-cycle discharge energy. The usable energy of a lithium-metal cell is controlled by a chain of coupled factors, including cathode structural stability, interfacial reactivity, electrolyte consumption, lithium inventory, transport kinetics and inactive component mass. High-voltage layered oxides require stable bulk and surface structures to preserve average voltage. Li-rich oxides need reversible oxygen redox and suppressed voltage decay. Sulfur and oxygen cathodes require reaction architectures that maintain high capacity under high-loading and lean-electrolyte conditions. Lithium-metal anodes require stable SEI formation and limited lithium excess. At the pouch-cell level, lightweight design and transparent reporting standards are essential for evaluating whether a battery chemistry can truly achieve high practical energy density.

Overall, future research should shift from maximizing material-level capacity to maximizing retained energy under realistic full-cell constraints. Only when cathode chemistry, electrolyte design, lithium-metal protection and cell-level mass management are optimized together can lithium-metal batteries move closer to practical high-energy applications.
